# In vivo and in vitro evaluation of combretastatin A-4 and its sodium phosphate prodrug

**DOI:** 10.1038/sj.bjc.6692174

**Published:** 1999-12

**Authors:** K Grosios, S E Holwell, A T McGown, G R Pettit, M C Bibby

**Affiliations:** Clinical Oncology Unit, University of Bradford, Bradford, West Yorkshire BD7 IDP, UK; CRC Paterson Inst for Cancer Research Christie Hospital NHS Trust, Manchester, UK; Cancer Research Institute and Department of Chemistry, Arizona State University, Tempe, AZ, USA

**Keywords:** combretastatin A-4, anti-vascular, orthotopic, colon tumour

## Abstract

The anti-tumour effects and mechanism of action of combretastatin A-4 and its prodrug, combretastatin A-4 disodium phosphate, were examined in subcutaneous and orthotopically transplanted experimental colon tumour models. Additionally, the ability of these compounds to directly interfere with endothelial cell behaviour was also examined in HUVEC cultures. Combretastatin A-4 (150 mg kg^–1^, intraperitoneally (i.p.)) and its water-soluble prodrug (100 mg kg^–1^, i.p.) caused almost complete vascular shutdown (at 4 h), extensive haemorrhagic necrosis which started at 1 h after treatment and significant tumour growth delay in MAC 15A subcutaneous (s.c.) colon tumours. Similar vascular effects were obtained in MAC 15 orthotopic tumours and SW620 human colon tumour xenografts treated with the prodrug. More importantly, in the orthotopic models, necrosis was seen in vascularized metastatic deposits but not in avascular secondary deposits. The possible mechanism giving rise to these effects was examined in HUVEC cells. Here cellular networks formed in type I calf-skin collagen layers and these networks were completely disrupted when incubated with a non-cytotoxic concentration of combretastatin A-4 or its prodrug. This effect started at 4 h and was complete by 24 h. The same non-cytotoxic concentrations resulted in disorganization of F-actin and β-tubulin at 1 h after treatment. In conclusion, combretastatin A-4 and its prodrug caused extensive necrosis in MAC 15A s.c. and orthotopic colon cancer and metastases, resulting in anti-tumour effects. Necrosis was not seen in avascular tumour nodules, suggesting a vascular mechanism of action. © 1999 Cancer Research Campaign


*British Journal of Cancer* (1999) **81**(8), 1318–1327
© 1999 Cancer Research Campaign
Article no. bjoc.1999.0847

### In vivo and in vitro evaluation of combretastatin A-4 and its sodium phosphate prodrug

**K Grosios[1], SE Holwell[1], AT McGown[2], GR Pettit[3] and MC Bibby[1]**

[1]Clinical Oncology Unit, University of Bradford, Bradford, West Yorkshire BD7 IDP, UK; [2]CRC Paterson Inst for Cancer Research Christie Hospital NHS Trust, Manchester, UK; [3]Cancer Research Institute and Department of Chemistry, Arizona State University, Tempe, AZ, USA

**Summary** The anti-tumour effects and mechanism of action of combretastatin A-4 and its prodrug, combretastatin A-4 disodium phosphate, were examined in subcutaneous and orthotopically transplanted experimental colon tumour models. Additionally, the ability of these compounds to directly interfere with endothelial cell behaviour was also examined in HUVEC cultures. Combretastatin A-4 (150 mg $kg^{-1}$, intraperitoneally (i.p.)) and its water-soluble prodrug (100 mg $kg^{-1}$, i.p.) caused almost complete vascular shutdown (at 4 h), extensive haemorrhagic necrosis which started at 1 h after treatment and significant tumour growth delay in MAC 15A subcutaneous (s.c.) colon tumours. Similar vascular effects were obtained in MAC 15 orthotopic tumours and SW620 human colon tumour xenografts treated with the prodrug. More importantly, in the orthotopic models, necrosis was seen in vascularized metastatic deposits but not in avascular secondary deposits. The possible mechanism giving rise to these effects was examined in HUVEC cells. Here cellular networks formed in type I calf-skin collagen layers and these networks were completely disrupted when incubated with a non-cytotoxic concentration of combretastatin A-4 or its prodrug. This effect started at 4 h and was complete by 24 h. The same non-cytotoxic concentrations resulted in disorganization of F-actin and β-tubulin at 1 h after treatment. In conclusion, combretastatin A-4 and its prodrug caused extensive necrosis in MAC 15A s.c. and orthotopic colon cancer and metastases, resulting in anti-tumour effects. Necrosis was not seen in avascular tumour nodules, suggesting a vascular mechanism of action. © 1999 Cancer Research Campaign

**Keywords**: combretastatin A-4; anti-vascular; orthotopic; colon tumour

Differences in the architecture, cellular and biochemical composition between normal and tumour blood vessels provide the basis for specific vascular targeting of tumours. The newly formed tumour vessels are usually thin-walled capillaries or sinusoids with little more than an endothelial lining backed by a basement membrane, and prone to spontaneous haemorrhage and thrombosis (Egawa et al, 1979; Bujaski et al, 1989). Tumour vessels also seem to lack smooth muscle and innervation, are often more permeable and tortuous, and exhibit variable diameters with suboptimal intercapillary spacing (Warren, 1979); characteristics which promote conditions of hypoxia and acidosis (Vaupel et al, 1989). The tumour vasculature contains vessels, particularly at the tumour margin, that are leaky to macromolecules (Dvorak et al, 1988) which, combined with poor lymphatic drainage, can lead to increased interstitial pressure, further impairing tumour perfusion (Vaupel et al, 1989). Currently, not all of these differences between tumour and normal vasculature are well understood. Nevertheless, the fact that vascular development is an absolute requirement for tumour growth and progression makes targeting of vasculature and inhibition of angiogenesis very important and promising anticancer strategies. Generally, in contrast to the antiproliferative effects of anti-angiogenic therapy, anti-vascular approaches aim to cause rapid and catastrophic shutdown in the vascular function of the tumour, leading to extensive secondary tumour cell death.

Various approaches have been employed in anti-vascular targeting in experimental cancer therapy. For example, manipulation of tumour blood flow by agents such as hydralazine and nicotinamide to improve the effectiveness of bioreductive agents administered alone or in combination with radiation and/or hyperthermia, has been described (Chaplin et al, 1991; Bibby et al, 1993). Targeting of tumour vasculature directly, was demonstrated using flavone acetic acid (FAA), a synthetic flavonoid, which has been shown to cause vascular shutdown and reduction in tumour blood flow in experimental tumours (Bibby et al, 1989*a*, 1991*a*). These effects were found to be mediated by the production of tumour recrosis factor α (TNF-α) (Mahadevan et al, 1990). Unfortunately, the very promising results of FAA against experimental tumours were not repeated in clinical trials. However the FAA analogue 5,6-dimethylxanthenone-4-acetic acid (5,6-MeXAA) (Rewcastle et al, 1989; Ching et al, 1992; Laws et al, 1995) is currently under investigation in clinical trial.

The anti-mitotic agent colchicine was amongst the first agents shown to possess anti-vascular activity, producing haemorrhagic necrosis in experimental tumours (Clearkin, 1937). Tubulin is also the site of action of the clinically used class of anticancer compounds, the vinca alkaloids. These compounds exert their anticancer effect by binding to tubulin and preventing its polymerization to form microtubules (Chabner and Collins, 1990), thus inhibiting a number of cellular processes, including mitosis. Taxol also interferes with microtubule dynamics by promoting the formation of highly stable microtubules which resist depolymerization. Binding of taxol alters the conformation of the tubulin subunit therefore greatly retarding tubulin heterodimer dissociation and promoting microtubule assembly (Schiff et al, 1979; Schiff and Horwitz, 1980).

*Received 10 September 1998*
*Revised 25 February 1999*
*Accepted 10 May 1999*

*Correspondence to:* MC Bibby

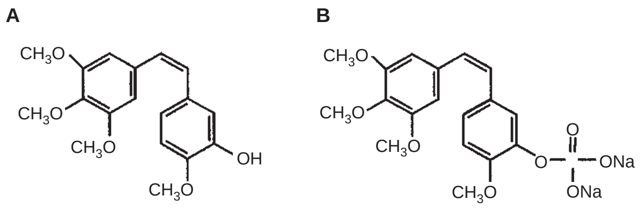

**Figure 1** The chemical structures of (**A**) combretastatin A-4 and (**B**) combretastatin A-4-disodium phosphate

Much interest has recently been generated in a series of stilbenes isolated from the South African tree *Combretum caffrum*. The most studied of these are combretastatin A-4, and its soluble phosphate prodrug (Pettit et al, 1989, 1995) (Figure 1). Combretastatin A-4 has been shown to bind to tubulin at the same site as colchicine does, but with even higher affinity (McGown and Fox, 1989; Pettit et al, 1989). Its prodrug, combretastatin A-4 disodium phosphate, has been shown to induce vascular shutdown in subcutaneous (s.c.) experimental tumours at doses less than one-tenth of the maximum tolerated dose (MTD) and to be more cytotoxic to rapidly proliferating rather than quiescent endothelial cells in vitro (Dark et al, 1997).

In this study initial experiments using a refractory s.c. murine adenocarcinoma (MAC 15A s.c.) were performed in order to confirm the previously reported effects of combretastatin A-4 and its prodrug on tumour growth delay at well-tolerated doses and to assess vascular damage in these tumours. Subsequent work concentrated on orthotopic murine (MAC 15) and human (SW620) colon adenocarcinomas, since the orthotopic site is thought by many to be more clinically relevant. In the MAC 15 orthotopic model, tumour growth in the colon is often accompanied by formation of metastasis in other parts of the body (Cowen et al, 1995). Therefore, the anti-tumour effects of combretastatin A-4 and its prodrug were assessed in the primary site and, where appropriate, in metastatic deposits. A mechanistic study to examine the ability of these compounds to directly interfere with human umbilical vein endothelial cells (HUVEC) and alter their growth properties and morphology in vitro was also carried out.

### MATERIALS AND METHODS

### Animals

NMRI mice (B&K Universal Ltd, Hull, UK) and nude-NCR-Nu mice (NCI, Frederick, MD, USA), aged 6–8 weeks were used. Mice received CRM diet (Special Diet Services, Witham, UK) and water ad libitum. All animal procedures were carried out under a project licence issued by the Home Office, London, UK and were performed according to UK guidelines (UKCCCR Guidelines, 1998).

### Subcutaneous tumours

MAC 15 is a moderately rapidly growing, well-differentiated, tubular adenocarcinoma originally induced in the colon of NMRI mice (Double et al, 1975). MAC 15A tumours are poorly differentiated with relatively fast growth rate and were produced by s.c. injection of MAC 15A cells from ascitic fluid. MAC 15A ascites tumour was derived by intraperitoneal (i.p.) implantation of MAC 15 tumour pieces, using the method described by Double and Cifuentes de Castro (1978). Human colon SW620 s.c. tumours were obtained from s.c. injections of the corresponding tumour cell line and passaged routinely in NCR-Nude mice.

### Orthotopic transplantation procedure

This was performed as previously described by Cowen et al (1995). The MAC 15 s.c. donor tumour was aseptically removed from the s.c. site, placed in a Petri dish containing sterile saline, penicillin and streptomycin (Sigma, Poole, UK), and the viable tissue was cut into 1-mm$^3$ pieces. The NMRI mice used had their abdomen shaved and were anaesthetized with Metophane inhalation anaesthetic (C-Vet, Leyland, UK). An incision was made on the lower left of the midline and the caecum was gently externalized. The piece of tumour was then placed on the surface of the caecum and covered with a small disc of cellulose acetate membrane (type RA- pore size 1–2 μm, Millipore UK Ltd, Watford, UK). The implant was fixed into position with Histoacryl blue tissue adhesive (Davis & Gek, Cyanamid, UK). The caecum was then returned to its normal position in the abdomen and the incision in the abdominal wall sealed using the tissue adhesive. Finally the skin was closed using autoclips, which were removed once sufficient wound healing had occurred. Orthotopic tumours usually developed 6–8 weeks after the transplantation. Identical procedures were carried out with SW620 human tumours in NCR mice.

### Compounds

Combretastatin A-4 (MW: 316) was dissolved in 10% dimethylsulphoxide (DMSO)/oil and combretastatin A-4 disodium phosphate (MW: 440) in saline. Both agents were administered i.p. in mice at 0.1 ml per 10 g of body weight.

### Assessment of tumour growth

Tumour volumes were assessed by caliper measurements of the perpendicular diameters and the volumes estimated using the equation: Volume = $(a^2 \times b)/2$ (Geran et al, 1972), where a is the smaller and b is the larger diameter of the two.

### Histological assessment

Tumours were collected and fixed in Bouin's fixative for 24 h. These were then dehydrated and embedded in paraffin. Sections were cut at 5-μm and stained using haematoxylin and eosin (H&E). A minimum of five sections from each tumour was examined. As the prodrug, rather than the lead compound, was selected for clinical evaluation the extent of necrosis in tumours from prodrug treated animals was quantifed and the percentage necrosis determined by image analysis (Seescan, Cambridge, UK) using the method described by Laws et al (1995).

### Hoechst-33342 studies

H-33342 (bisBenzimide, Sigma) was dissolved in sterile saline and administered at 40 mg kg$^{-1}$ intravenously (i.v.) (volume 5 ml kg$^{-1}$) (Smith et al, 1988). One minute after injection the mouse was killed by cervical dislocation, dissected and the tumour

© 1999 Cancer Research Campaign 

resected. Tumours were wrapped in aluminium foil and immediately immersed in liquid nitrogen for subsequent frozen sectioning. Frozen sections (5-µm) were cut on a cryostat (Bright Instrument Co. Ltd, Huntingdon, UK) and viewed at ×25 objective magnification on a light microscope using incident UV illumination. Perfusion was observed as fluorescence and counts were performed, where squares containing fluorescence, in a graticule with a grid of 400 squares, were counted as positive. A minimum of five sections per tissue was examined and five fields counted per section.

### Statistical analysis of data

All the numerical data obtained from chemotherapy experiments, cytotoxicity assays, necrosis analysis or fluorescent dye staining are given as the mean values ± standard deviation (s.d). Statistical analysis of chemotherapy and image analysis results was carried out using the Mann–Whitney *U*-test or Student's *t*-test.

### HUVEC culture

All cell culture media and reagents were purchased from Sigma. HUVEC were isolated according to the method described by Jaffe et al (1973). Umbilical cords (~20 cm) from elective Caesarean sections were received from the Maternity Unit of Bradford Royal Infirmary. Isolated HUVEC were grown in medium M199 supplemented with 10% human serum and 100 mM sodium pyruvate, 200 mM L-glutamine, 5000 IU $ml^{-1}$ penicillin and 5000 µg $ml^{-1}$ streptomycin. All culture flasks used had been previously coated with 0.2% gelatin in phosphate-buffered saline (PBS).

### *HUVEC network formation assay*

Type I calf-skin collagen solution (900 µl of 1.1 mg $ml^{-1}$ in 0.01 N acetic acid) was mixed with 100 µl of 10× (ten times concentrated) MEM and 150 µl of freshly prepared 7.5% (w/v) sodium bicarbonate solution. This collagen solution (300 µl) was then added to each well, in 24-well plates and incubated at 37°C until the gels set (approximately 15–20 min). Pre-warmed culture medium was then added to wells and the gels incubated for 3 h to equilibrate. After the 3-h incubation period the medium was removed and replaced with 0.5 ml of fresh pre-warmed medium, containing $1 \times 10^5$ cells $ml^{-1}$ and the gels were incubated for a further 24 h (37°C, 5% carbon dioxide). The medium was then carefully removed and a second layer of collagen, of the same composition as the first one, was added and left to set before addition of fresh pre-warmed medium. The following day HUVEC networks could be observed.

### *HUVEC migration assay*

The ability of HUVEC to migrate through collagen was examined using Transwell chambers (Costar, High Wycombe, UK). The membrane of the upper chamber of transwells was coated with 100 µl of the same collagen solution as that used in the tube formation assays was left at 37°C for approximately 20 min to gel. Before being used gels were incubated in medium for 3 h. Cells ($5 \times 10^3$) were then seeded on the gel and left at 37°C for 2 h before the media in the wells were replaced with either fresh medium or medium containing non-cytotoxic concentration of the test compound (combretastatin A-4 or A-4 phosphate). Following 24-h incubation at 37°C, the transwell was removed and fixed for 2 h in Bouin's solution. The filters were then removed, stained with H&E and microscopically examined for the presence of HUVEC. Each exposure was performed at least in triplicate.

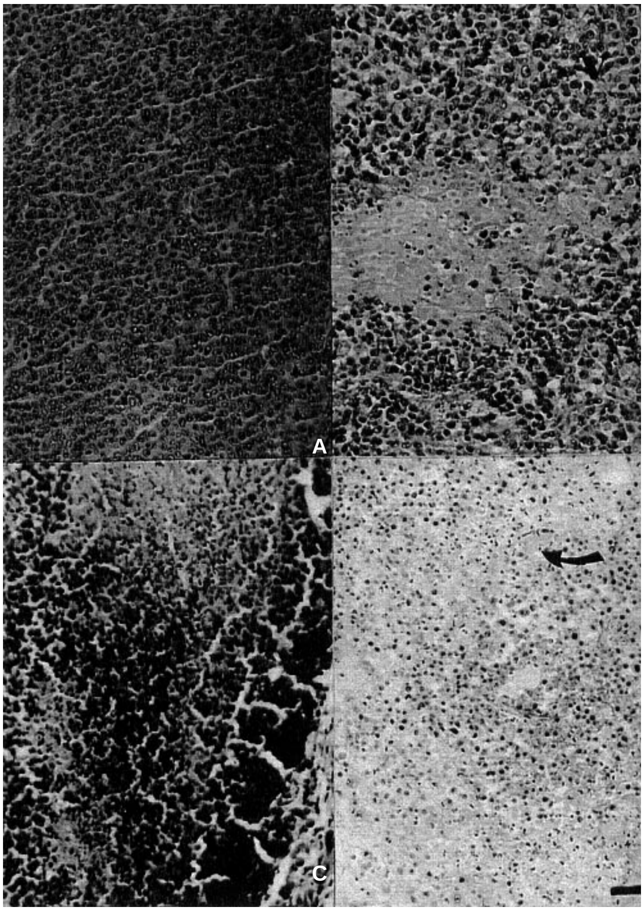

**Figure 2** Representative sections showing the morphological appearance (H&E staining) of MAC 15A s.c. tumours in NMRI mice treated with single i.p. dose combretastatin A-4 (150 mg $kg^{-1}$), (**A**) control, (**B**) 2 h, (**C**) 4 h, (**D**) 24 h. V: viable tumour area, arrow: area of necrosis (Bar: 30 µm)

### *HUVEC neutral red assay*

The neutral red (NR) assay was performed as described by Borenfreund et al (1990). Gelatinized, flat-bottom, 96-well plates (Costar) were seeded with $4 \times 10^3$ cells per well and incubated for 18 h at 37°C. The cells were then incubated with drug in culture medium or DMSO at 37°C, 5% carbon dioxide, for the appropriate time. After this the cells were washed with Hank's balanced saline solution (HBSS) and the medium was replaced with medium containing NR dye (40 µg $ml^{-1}$), incubated for 3 h following which the cells were fixed by adding 100 µl of 0.5% formalin–calcium chloride to each well. The dye was extracted by adding 200 µl of 1% acetic acid/50% ethanol per well and the absorbance determined at 540 nm using an enzyme-linked immunosorbent assay (ELISA) spectrophotometer (Multiscan Plus, Labsystems, Life Sciences International Ltd, Basingstoke, UK). Each experiment was performed at least in triplicate. A calibration curve which illustrated linearity between cell number and absorbance at 540 nm was also performed.

 *© 1999 Cancer Research Campaign*

**Table 1** The ability of combretastatin A-4* or the prodrug** to produce a growth delay was examined in NMRI mice bearing subcutaneous MAC 15A murine colon adenocarcinoma

| Dose (mg kg$^{-1}$) | Mean time to RTV2 (±s.d.) (days) | Median time to RTV2 (days) | Growth delay (days) | Significance |
|---|---|---|---|---|
| 50* | 1.45 (± 0.66) | 1.52 | None | NS |
| 100* | 2.96 (± 0.70) | 3.20 | 1.70 | $P < 0.05$ |
| 200* | Toxic | | | |
| Control | 1.89 (± 0.30) | 1.50 | | |
| 100** | 3.83 (± 1.19) | 3.83 | 1.53 | $P < 0.05$ |
| Control | 2.40 (± 0.77) | 2.30 | | |

Treatment began when tumours had reached a minimum size of 4 × 5 mm and effect of therapy was monitored by daily caliper measurements of the tumour ($n \geq 6$). Semi-log plots of relative tumour volume against time were made and time taken for tumour-doubling to occur was recorded. Significance of growth delay was determined by a Mann–Whitney *U*-test comparing doubling time in control versus treated tumours.

**Table 2** Percentage area of necrosis in sc MAC 15A tumours 24 h after treatment with combretastatin-A4 prodrug as determined by image analysis

| Treatment | Area of necrosis (mean % ± s.d.) |
|---|---|
| 100 mg kg$^{-1}$ | 91.74 ± 10.51 |
| Control | 10.53 ± 16.95 |

$P < 0.001$.

**Table 3** Percentage area of necrosis in orthotopically transplanted MAC 15 tumours 24 h after treatment with combretastatin-A4 prodrug as determined by image analysis

| Treatment | Area of necrosis (mean % ± s.d.) |
|---|---|
| 100 mg kg$^{-1}$ | 97.34 ± 9.99 |
| Control | 4.21 ± 6.74 |

$P < 0.001$.

### *F-actin staining*

Staining of cytoskeletal F-actin was achieved using rhodamine conjugated phalloidin. HUVEC were grown on thermanox coverslips (ICN Biomedicals Inc, Oxton, UK). Following treatment with the test compound the coverslips were washed with PBS, permeabilized in 0.5% Triton X-100 in PBS, fixed in 1.5% formaldehyde and washed in PBS. Coverslips were incubated for 1 h in the rhodamine–phalloidin solution (1 μg ml$^{-1}$), rinsed in PBS and mounted using 80% glycerol in PBS. The coverslips were then microscopically examined under fluorescence. Untreated controls were also processed in the same way.

### *Tubulin staining*

Tubulin staining was performed using a three-step indirect immunoperoxidase technique. HUVEC were grown on thermanox coverslips and, following treatment with the test compound, were washed in PBS, permeabilized in 0.5% Triton X-100 in PBS and fixed in 1.5% formaldehyde in PBS for 1 h. After washing in PBS the coverslips were incubated in a humid chamber with the primary antibody (mouse monoclonal anti-β tubulin antibody, 1:400 dilution, Amersham International plc, Little Chalfont, UK) for 2 h. Following this, the coverslips were washed in PBS and incubated with the secondary antibody (rabbit anti-mouse biotinylated, Dako, High Wycombe, UK) for 45 min. The coverslips were then washed again in PBS and incubated with avidin–biotin–peroxidase complex (Dako) for 30 min. Visualization was carried out using the chromagen 3,3′-diaminobenzidine tetrahydrochloride (Sigma) and enhancement was with copper sulphate solution for 5 min. Coverslips were finally counterstained with haematoxylin, dehydrated and mounted with DPX. Control coverslips were processed by the same procedure.

### RESULTS

### In vivo studies

### *The effects of combretastatin A-4 on MAC 15A s.c. tumour growth*

The ability of combretastatin A-4 to cause tumour growth delay was assessed in MAC 15A s.c. tumours. Treatment began when tumours could be reliably measured by calipers, i.e. when tumours had reached minimum diameters of 4 × 5 mm. Initial treatment of mice bearing such tumours with single i.p doses of combretastatin A-4 (100 mg kg$^{-1}$) resulted in a modest tumour growth delay of 1.7 days ($P < 0.05$) (Table 1). Treatment with 50 mg kg$^{-1}$ did not induce a growth delay. Subsequent experiments with a higher dose of combretastatin A-4 (200 mg kg$^{-1}$) in the same tumour model did not further increase tumour growth delay but did cause toxicity with three out of five mice having to be killed. Two mice were killed, one on day 2 and the other on day 3. Further studies with the water-soluble prodrug (100 mg kg$^{-1}$) produced similar anti-tumour effects (Table 1). A further experiment in which mice were treated with the same dose of the prodrug on day 1 after tumour cell inoculation (prior to vascularization) resulted in no measurable tumour growth delay.

### Morphological studies in 15A s.c. models

The effects of combretastatin A-4 and its prodrug, combretastatin A-4 disodium phosphate, on tumour morphology were assessed in NMRI mice bearing MAC 15A s.c. tumours. Four mice were treated with 150 mg kg$^{-1}$, single i.p dose (the dose that was found to cause tumour growth delay with no obvious toxicity) of combretastatin A-4. A control group of four mice was injected with the solvent. Animals were killed 24 h after treatment, their tumours removed, fixed, processed and stained and a number of sections from each tumour was examined. All MAC 15A s.c. tumours from untreated animals showed their usual histological appearance of poor differentiation and limited necrosis (Figure 2A). In contrast, all the tumours from treated mice showed very extensive areas of haemorrhagic necrosis. Some small areas of viable tumour could also be found, particularly in the tumour periphery. The time taken for the haemorrhagic necrosis to occur was also investigated. A number of sections from MAC 15A s.c. tumours excised from groups of four treated and four control NMRI mice were examined at 1, 2 and 4 h after treatment. Following a single i.p dose of 150 mg kg$^{-1}$ combretastatin A-4 or 100 mg kg$^{-1}$ of the prodrug, the first signs of haemorrhagic necrosis appeared 2 h after the injection. No necrosis was seen at 1 h but tumours appeared extensively necrotic 4 h after the

© 1999 Cancer Research Campaign 

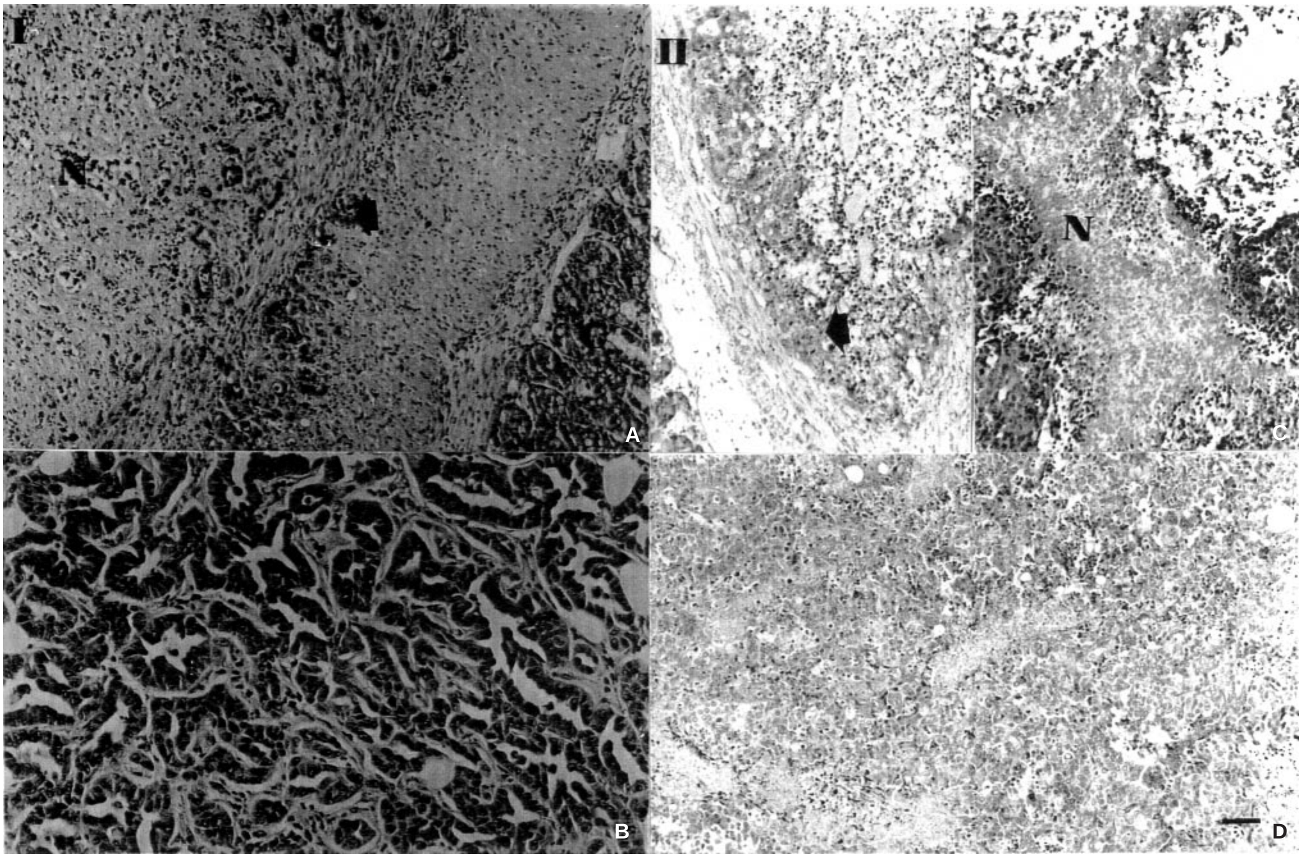

**Figure 3** Representatative sections showing the morphological appearance (H&E staining) of panel I (left) MAC 15, and panel II (right) SW 620, orthotopic tumour in mice (**A** and **C**) twenty-four hours after treatment with single i.p. dose of combretastatin A-4 (150 mg $kg^{-1}$) for MAC 15 or combretastatin A-4 phosphate (100 mg $kg^{-1}$) for SW620. (**B** and **D**) tumours from control animals. Arrow: viable tumour, N: necrosis, C: normal colon (Bar: 30 μm)

injection (Figure 2 B,C) The massive necrosis resulting 24 h after treatment with combretastatin A-4 is demonstrated in Figure 2D.

In order to quantify the extent of necrosis produced by the prodrug, sections from the tumours removed 24 h after treatment underwent image analysis. This analysis demonstrated massive necrosis in treated tumours (Table 2). Similar morphological changes were seen following treatment of NCR-Nu (immunocompromised) mice bearing MAC 15A s.c. tumours with 150 mg $kg^{-1}$ combretastatin A-4 and 100 mg $kg^{-1}$ combretastatin A-4 phosphate.

### Orthotopic model studies

The antitumour effects of combretastatin A-4 and its phosphate derivative were also assessed in the MAC 15 orthotopic tumour model. After MAC 15 orthotopic transplantation, the 'primary' adenocarcinoma development was often followed by development of tumour metastatic deposits in the lung, lymph nodes, liver and other sites. Unfortunately, assessment of the development of metastasis is possible only after the animal has been killed. Five NMRI mice bearing MAC 15 orthotopic tumours were treated with a single i.p. dose of 150 mg $kg^{-1}$ and another three NMRI mice bearing MAC 15 orthotopic tumours were treated with a single i.p. dose of 100 mg $kg^{-1}$ combretastatin A-4 disodium phosphate. These doses having been previously demonstrated to cause haemorrhagic necrosis in the s.c. model. Twenty-four hours after treatment the tumours were removed and microscopic examination of a number of sections from each tumour showed that extensive haemorrhagic necrosis was present in the 'primary', orthotopic tumour. As can be seen in Figure 3, the normal colon appears unaffected. Again, however, areas of viable tumour could also be seen in the tumour periphery. Tumours from control animals and those treated with the prodrug were analysed for extent of necrosis. As can be seen from Table 3 the effects of the prodrug are very marked. Orthotopic tumours removed from two animals treated with 75 mg $kg^{-1}$ combretastatin A-4 phosphate were very similar in appearance with extensive haemorrhagic necrosis.

In addition to the MAC 15 orthotopic tumours, two NCR-Nu mice bearing SW 620 orthotopic human colon tumours were treated with 100 mg $kg^{-1}$, single i.p. dose of combretastatin A-4 phosphate. The same effects as those seen in the treated MAC 15 orthotopic tumours (Figure 3) were also observed in all the sections examined from these tumours. Although insufficient samples were available for absolute quantification, in this case very extensive haemorrhagic necrosis was seen in treated but not control mice. Again a rim of viable tumour was observed in the periphery.

One important characteristic of the MAC 15 orthotopic tumour model is that the primary tumour as well as some of the metastatic deposits (including body wall, axillary lymph node and kidney deposits) (Figure 4) develop extensive vasculature. In contrast, deposits that form in the lungs of the animal remain relatively small in size and avascular (Figure 5).

The profound effects of these compounds on the primary

 *© 1999 Cancer Research Campaign*

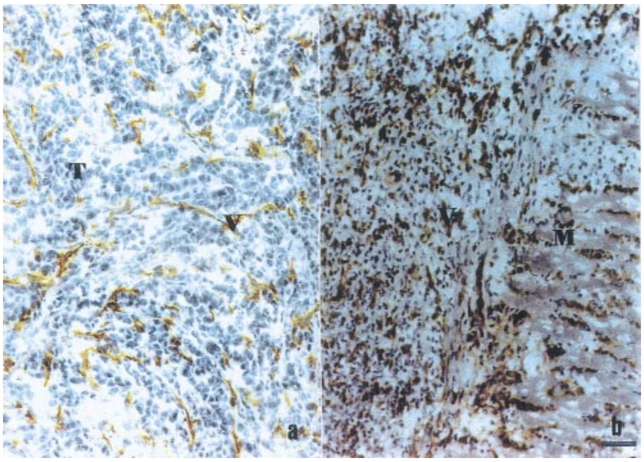

**Figure 4** (**A**) Immunostaining of frozen section of MAC 15 tumour using an antibody to CD31, showing blood vessel development in the tumour in the orthotopic site in NMRI mouse. (**B**) Immunostaining of frozen section of body wall deposit developing following MAC 15 orthotopic transplantation in NMRI mouse. V: blood vessel, T: tumour, M: muscle (Bar: 30 μm)

tumours were also accompanied by effects on metastatic tumour deposits. Liver deposits were not found in any of the mice treated, but tumour deposits were excised from the abdominal wall (in three animals), kidney (in one animal) and lymph nodes (in two animals) of treated mice. Microscopic examination of a number of sections from all of these deposits showed that they all exhibited large areas of necrosis (Figure 6A, kidney).

Three of the mice treated with combretastatin A-4 and three of the mice treated with combretastatin A-4 disodium phosphate had developed avascular tumour metastatic deposits in their lungs. These remained unaffected by the treatment, i.e. no signs of necrosis were seen in any of the deposits (Figure 6B).

### Vascular shutdown studies

Vascular shutdown studies using Hoechst 33342 fluorescent dye showed that treatment with a single i.p. dose of 100 mg $kg^{-1}$ of combretastatin A-4 disodium phosphate resulted in almost complete vascular shutdown in both MAC 15A s.c. and MAC 15 orthotopic tumours, 4 h after treatment The same results were also obtained in MAC 15A s.c. tumours, 4 h after treatment with a single i.p. dose of 150 mg $kg^{-1}$ of combretastatin A-4 (in all three cases four tumour-bearing mice were treated and another four were used as controls). The vascular counts for untreated MAC 15A s.c. and MAC 15 orthotopic tumours were 47.56 (s.d. 7.94) and 69.97 (s.d. 16.43) respectively, whereas in all treated tumours very limited fluorescence could be seen, again only in the tumour periphery.

### In vitro studies

Initial experiments were performed using the NR assay in order to establish non-cytotoxic concentrations of these compounds. Employing 24-h exposure to 0.31 μM and 0.40 μM of combretastatin A-4 and combretastatin A-4 disodium phosphate respectively resulted in less than 40% of HUVEC cell death, i.e. 68.3% (s.d. 6.87) HUVEC viability after exposure to combretastatin A-4 and 63.1% (s.d. 2.82) cell viability after exposure to combretastatin A-4-phosphate. At the same concentrations and exposure time the percentage cell survival in a 6-day assay was approximately 80% (85.9%, s.d. 8.90 for combretastatin A-4, and 83.5 s.d. 5.62 for A-4 phosphate).

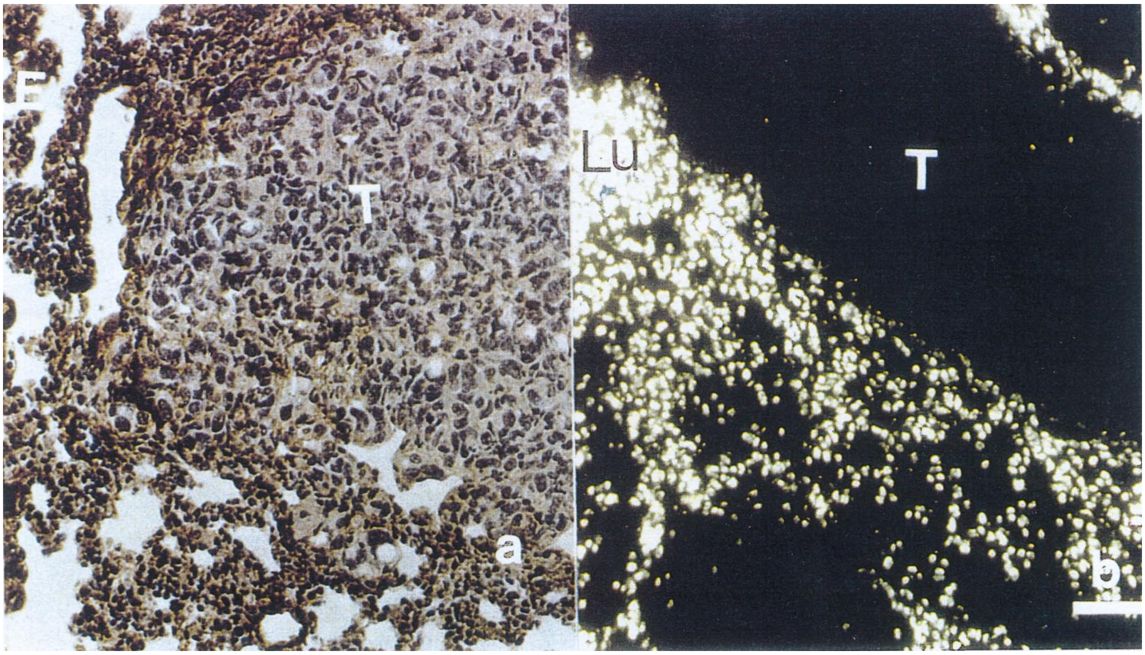

**Figure 5** Frozen section of tumour deposit formed in the lung of NMRI mouse following MAC 15 orthotopic transplantation showing the absence of blood vessel development. (**A**) Immunostaining using an antibody to von Willebrand factor and (**B**) Hoechst 33342 fluorescent dye staining. T: tumour deposit, Lu: lung, (Bar: 30 μm)

*© 1999 Cancer Research Campaign* 

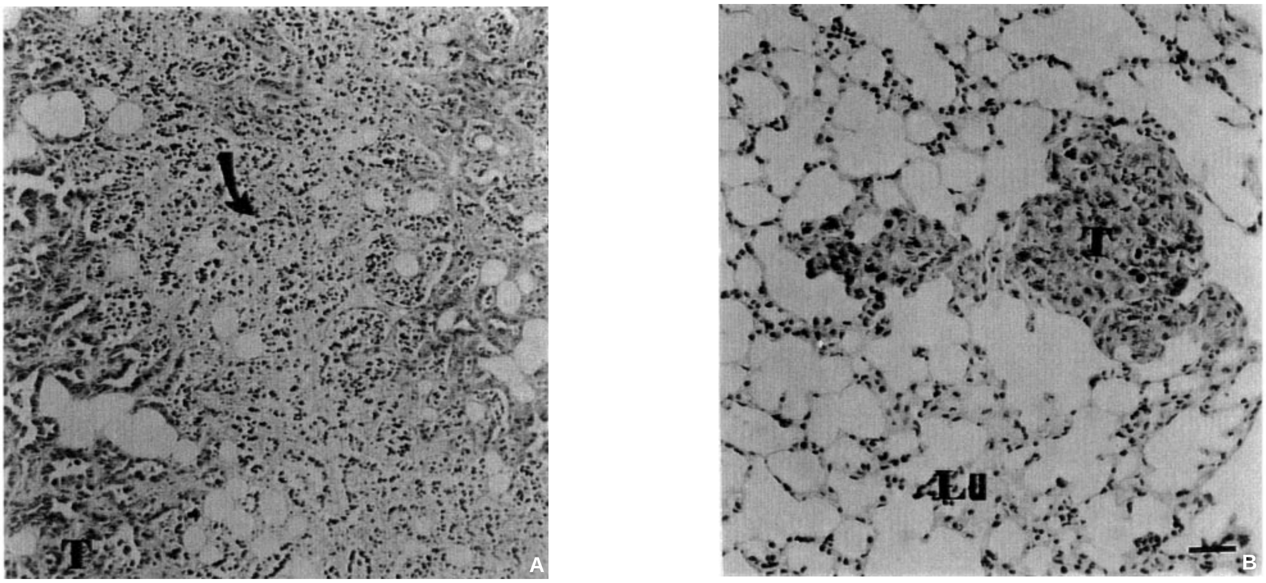

**Figure 6** Morphological appearance (H&E staining) of metastatic tumour deposits in (**A**) kidney, (**B**) lung, of NMRI mice bearing MAC 15 orthotopic tumours and treated with single, i.p. dose combretastatin A-4 (150 mg $kg^{-1}$). Arrow: necrotic area, T: viable tumour, Lu: lung (Bar: 30 μm)

### Effects on HUVEC networks in collagen

Addition of media containing either 0.31 μM of combretastatin A-4 or 0.40 μM combretastatin A-4 disodium phosphate (non-cytotoxic concentrations) to already established endothelial cell networks in type I calf-skin collagen resulted in rapid disruption of these networks (Figure 7). Disruption started after approximately 4 h and was complete after 24 h of exposure to the compounds. These exposures were performed at least in triplicate and the same degree and time of appearance of network disruption was seen in all the exposed wells. In addition, the same non-cytotoxic concentrations of either 0.31 μM of combretastatin A-4 or 0.40 μM combretastatin A-4 disodium phosphate inhibited migration of HUVEC through type I calf-skin collagen in transwell chambers (results not shown).

### Effects on HUVEC cytoskeleton

Treatment of HUVEC with non-cytotoxic doses of combretastatin A-4 or combretastatin A-4 disodium phosphate (0.31 μM and 0.40 μM respectively) resulted in complete cytoskeletal disorganization and disruption of both F-actin filaments and tubulin microtubules (Figure 7). This disruption was seen approximately 1 h after treatment and was uniform across the cell monolayer on the coverslip.

### DISCUSSION

The main aim of this study was to investigate the role of vasculature in the anti-tumour response of a novel compound, combretastatin A-4 and its water-soluble disodium phosphate derivative, and to provide further insights into their mechanism of action. This study, has demonstrated major effects of combretastatin A-4 and its prodrug in human and murine tumours in clinically relevant sites. Experiments with both the parent compound and its prodrug had previously shown that they caused an increase in vascular resistance by a factor of at least 3 in rat P22 tumour perfused ex vivo. In addition, treatment with the prodrug resulted in haemorrhagic necrosis in CaNT and MDA-MB-231 tumours (Dark et al, 1997). We also found that treatment of MAC 15A s.c. tumours with combretastatin A-4 and its water-soluble prodrug caused tumour growth delay, which was accompanied by vascular shutdown 4 h after treatment. In addition, extensive haemorrhagic necrosis was seen with both combretastatin A-4 and A-4 phosphate, the appearance of which started 2 h after treatment and by 24 h was very extensive.

Extensive haemorrhagic necrosis was also observed in orthotopic MAC 15 tumours and the orthotopic human colon tumour xenograft SW620 at 24 h (the time point examined). Hoechst 33342 studies in the treated MAC 15 orthotopic tumours showed the presence of almost complete vascular shutdown 4 h after treatment. At these dose levels neither the parent compound nor its prodrug affected the normal colon, indicating possible tumour specificity. In the treatment of any disease, compounds are required that can achieve their effects at doses much lower than the MTD. Extensive necrosis was observed in orthotopic MAC 15 tumours treated with 100 mg $kg^{-1}$ and also doses down to 75 mg $kg^{-1}$ of combretastatin A-4 disodium phosphate. Dark et al (1997) described similar effects in s.c. tumours treated with doses of combretastatin A-4 disodium phosphate that were less than 10% of the MTD. This could prove an important aspect in the clinical evaluation of combretastatin A-4 prodrug as anti-vascular effects may occur well below the MTD in humans. Taxol exerts its effects at doses close to MTD (Rowinsky et al, 1990) and the same is true for other compounds known to interfere with vasculature, such as colchicine, vinblastine (which also binds tubulin), FAA and 5,6-MeXAA (Bibby et al, 1989*a*; Baguley et al, 1991). The studies described indicate that use of combretastatin A-4 may exert a more selective action against tumour vasculature than other anti-microtubule agents.

The fact that immune status can influence response to anti-tumour agents has been shown with FAA (Bibby et al, 1991*b*). However, treatment of MAC 15A s.c. tumours in NCR-Nu mice with combretastatin A-4 or its phosphate prodrug exhibited the same marked necrosis to that observed in NMRI mice (data not

 © 1999 Cancer Research Campaign

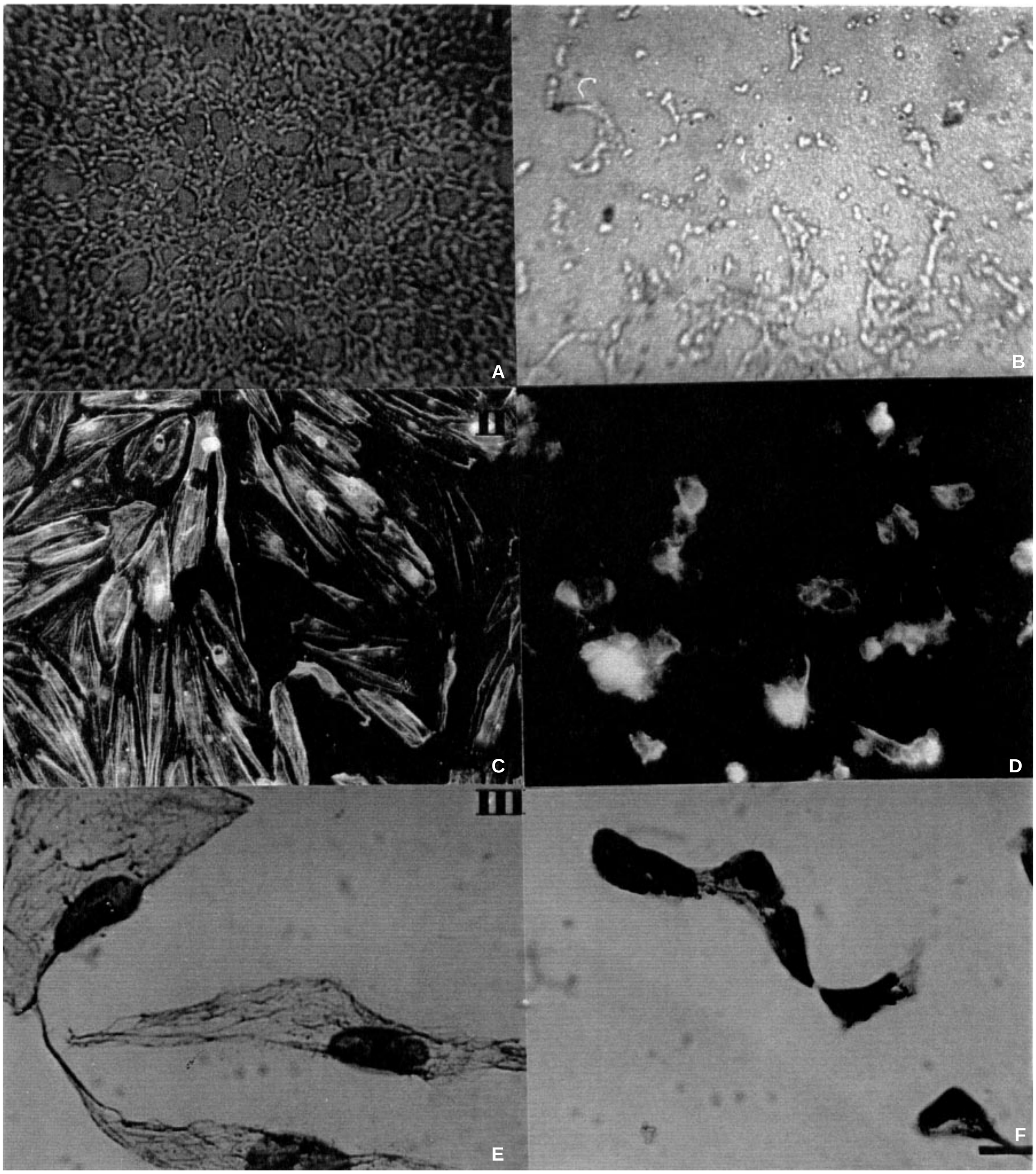

**Figure 7** Panel I (top): appearance of HUVEC networks in type I calf skin collagen, 4 h after treatment. (**A**) Control (incubated with media containing 1% DMSO), (**B**) treated with combretastatin A-4 (0.31 μM), (Bar: 60 μm). Panel II (middle): appearance of HUVEC cytoskeleton showing F-actin, (**C**) untreated, (**D**) 1 h after treatment with 0.31 μM combretastatin A-4 (Bar: 15 μm). Panel III (bottom): appearance of HUVEC cytoskeleton, showing β-tubulin, (**E**) untreated, (**F**) 1 h after treatment with 0.31 μM combretastatin A-4 (Bar: 15 μm)

shown) as did SW620 tumours indicating that this is unlikely to be a contributory factor for these compounds.

The anti-vascular effects of agents such as FAA have been attributed in part to their ability to induce TNF-α release (Mahadevan et al, 1990), whereas anti-vascular effects seen with some tubulin-binding agents do not involve TNF-α (Hill et al, 1995). Dark et al (1997) also reported, but no data were presented, that these effects of combretastatin A-4-phosphate do not involve TNF-α. Work in our laboratory failed to detect TNF-α in the serum of mice bearing tumours responding to combretastatin A-4.

Since tumour metastasis is a major problem in cancer therapy, compounds that are able to specifically affect the viability of metastases would prove very useful. The necrosis in metastatic deposits caused by combretastatin A-4 and its prodrug in this study at well-tolerated doses suggests that the water-soluble prodrug might be clinically useful. To date, the observation that haemorrhagic necrosis occurs only in vascularized tumour deposits is the clearest evidence that a direct effect of these compounds on tumour vasculature is essential for anti-tumour activity in vivo. It might be assumed that avascular deposits are unlikely to receive such high concentrations of drug as those with a well developed blood supply thereby reducing anti-tumour

*© 1999 Cancer Research Campaign* 

effects, but previous data have shown avascular MAC15A lung metastases to be equally as sensitive to 5-fluorouracil, chlorambucil and tauromustine as well-vascularized s.c. MAC15A tumours (Bibby et al, 1989*b*).

The ideal anti-vascular agent might have the ability to exert potent effects on both tumour cells as well as endothelial cells, e.g. vinblastine (Hill et al, 1995). Preferably it should exert its immediate effects on *dividing* endothelial cells, the cell type that it comes into direct contact with from the blood. The use of HUVEC cultures provided further insights into the mechanism of action of these two combretastatins and their ability to influence endothelial cell behaviour. Endothelial cells, including HUVEC, form cell networks when grown on specific substrata such as fibronectin, matrigel and type I collagen (Schor et al, 1983). This ability depends on cell density and electron microscopy studies have shown that the tubular networks that are formed consist of cells, held together by junctional complexes. They display a well-oriented cell polarity and form a lumen (Maciag et al, 1982; Montesano et al, 1983).

In this study, non-cytotoxic concentrations of combretastatin A-4 and the disodium phosphate derivative caused relatively rapid disruption of HUVEC networks and inhibited HUVEC migration through type I collagen. This implies that the anti-tumour effect may be brought about by the direct action of these agents on endothelial cells. Although it is likely that this is a feature of tubulin-binding drugs, in general these effects are seen with combretastatin-A4 at concentrations that are well below those that are cytoxic to endothelial cells and other cell types (Dark et al, 1997). F-actin and β-tubulin staining studies indicated that these effects result from the ability of combretastatin A-4 to bind to tubulin and cause complete cytoskeletal disorganization in endothelial cells. If this happened in vivo it may lead to collapse of the tumour vasculature. Both actin and tubulin appeared distorted in HUVEC after approximately 1 h of exposure which correlates with and may explain the timing of the appearance of haemorrhagic necrosis and vascular shutdown in vivo. Since combretastatin A-4 binds to tubulin, it is likely that tubulin disorganization is immediately followed by actin disorganization. These two cytoskeletal components are in very close association and apparently once one of them is disturbed the other one will not remain unaffected and would not be able to maintain cellular architecture (Birchmeier, 1984). Vero cells (African Green monkey kidney cells) have exhibited the same tubulin disorganization following treatment with combretastatin, and treatment of HUVEC with a similar compound, combretastatin A-1 resulted in the same tubulin disorganization as A-4 (Woods et al, 1995).

In conclusion, combretastatin A-4 and its disodium phosphate prodrug cause vascular shutdown and haemorrhagic necrosis in vascularized but not in avascular tumours in mice. These effects were observed not only in MAC 15A s.c. tumours and orthotopic MAC 15 and SW620 tumours in a clinically relevant site, but also in metastases. The in vitro studies indicate that these compounds do have the ability to interfere with endothelial cell behaviour and this might be the way they exert their actions. It is important that the clinical evaluation of combretastatin A-4-disodium phosphate be accompanied by appropriate investigation of anti-vascular effects.

### ACKNOWLEDGEMENTS

This work was supported by War on Cancer. The authors would also like to acknowledge Dr Dai Chaplin of the Gray Laboratory for helpful discussions and sharing data and the excellent support given by staff at the Maternity Unit at Bradford Royal Infirmary. MCB and ATM are members of the EORTC Screening and Pharmacology Group.

### REFERENCES

Baguley BC, Holdaway KM, Thomsen LL, Zhuang L and Zwi LJ (1991) Inhibition of growth of colon 38 adenocarcinoma by vinblastine and colchicine: Evidence for a vascular mechanism. *Eur J Cancer* **27**: 482–487

Bibby MC, Double JA, Loadman PM and Duke CV (1989*a*) Reduction of tumour blood flow by flavone acetic acid: a possible component of therapy. *J Natl Cancer Inst* **81**: 216–220

Bibby MC, Phillips RM and Double JA (1989*b*) Influence of site on the chemosensitivity of transplantable murine colon tumours to flavone acetic acid (LM975, NSC 347512). *Cancer Chemother Pharmacol* **24**: 87–94

Bibby MC, Double JA, Phillips RM and Quinn PK (1991*a*) Flavone acetic acid: is vascular shutdown the crucial mechanism of action. *Int J Radiat Biol* **60**: 395–399

Bibby MC, Phillips RM, Double JA and Pratesi G (1991*b*) Anti-tumour activity of flavone acetic acid (NSC 347512) in mice – influence of immune status. *Br J Cancer* **63**: 57–62

Bibby MC, Sleigh NR, Loadman PM and Double JA (1993) Potentiation of EO9 antitumour activity by hydralazine. *Eur J Cancer* **29A**: 1033–1035

Birchmeier W (1984) Cytoskeleton, structure and function. *Trends Biochem Sci* **9**: 192–195

Borenfreund E, Babich H and Martin-Alguacil N (1990) A rapid chemosensitivity assay with human normal and tumour cells in vitro. *In vitro Cell Dev Biol* **26**: 1030–1034

Bujaski A, Nowgrodzka Zagorska M, Lenko J and Miodonski AJ (1989) Angiomorphology of human renal cell carcinoma. *Virchows Archiv A Pathol Anat* **415**: 103–113

Chabner BA and Collins JA (1990) *Cancer Chemotherapy: Principles and Practice*. JB Lippincott: Philadelphia

Chaplin DJ, Peters CE, Horsman MR and Trotter MJ (1991) Drug induced perturbations in tumour blood flow: therapeutic potential and possible limitations. *Radiother Oncol* **20**: 93–101

Ching LM, Joseph WR and Baguley BC (1992) Antitumour responses to flavone-8-acetic acid and 5,6-dimethylxanthenone-4-acetic acid in immune deficient mice. *Br J Cancer* **66**: 128–130

Clearkin PA (1937) The effect of colchicine on normal and neoplastic tissues in mice. *J Pathol Bact* **44**: 469–480

Cowen SE, Bibby MC and Double JA (1995) Characterisation of the vasculature within a murine adenocarcinoma growing in different sites to evaluate the potential of vascular therapies. *Acta Oncol* **34**: 357–360

Dark GG, Hill SA, Prise VE, Tozer GM, Pettit GR and Chaplin DJ (1997) Combretastatin A-4, an agent that displays potent and selective toxicity towards tumour vasculature. *Cancer Res* **57**: 1829–1834

Double JA and Cifuentes de Castro L (1978) Chemotherapy of transplantable adenocarcinomas of the colon in mice. II. Development and characterisation of an ascitic cell line. *Cancer Treat Rep* **62**: 85–90

Double JA, Ball CR and Cowen PN (1975) Transplantation of adenocarcinomas of the colon in mice. *J Natl Cancer Inst* **54**: 271–275

Dvorak HF, Nagy JA, Dvorak JT and Dvorak AM (1988) Identification and characterization of the blood vessels of solid tumours that are leaky to circulating macromolecules. *Am J Pathol* **133**: 95–109

Egawa J and Ishioka K (1979) Construction of blood vessels in experimental tumours – some considerations on radioresistance In: *Treatment of Radioresistant Cancers,* Abe M, Sakamoto K and Phillips TL (eds), pp. 213–227. Elsevier/North Holland Biomedical Press: Amsterdam

Geran RI, Greenberg NH and MacDonald MM (1972) Protocols for screening chemical agents and natural products against tumours and other biological systems (Third edition). *Cancer Chemother Rep* **3**: 1–103

Hill SA, Sampson LE and Chaplin DJ (1995) Anti-vascular approaches to solid tumour therapy: evaluation of vinblastine and flavone acetic acid. *Int J Cancer* **63**: 119–123

Jaffe E, Nachman R, Becker C and Minick C (1973) Culture of human endothelial cells derived from umbilical veins. *J Clin Invest* **52**: 2745–2756

Laws AL, Matthew AM, Double JA and Bibby MC (1995) Preclinical in vitro and in vivo activity of 5,6-dimethylxanthenone-4-acetic acid. *Br J Cancer* **71**: 1204–1209

 © 1999 Cancer Research Campaign

McGown AT and Fox BW (1989) Structural and biochemical comparison of the anti-mitotic agents cholchicine, combretastatin A-4 and amphethinile. *Anti-Cancer Drug Des* **3**: 249–254

Maciag T, Kadish J, Wilkins L, Stemerman MB and Weinstein R (1982) Organizational behaviour of human umbilical vein endothelial cells. *J Cell Biol* **94**: 511–520

Mahadevan V, Malik STA, Meager A, Fiers W, Lewis GP and Hart IR (1990) Role of tumour necrosis factor in flavone-acetic acid-induced tumour vasculature shutdown. *Cancer Res* **50**: 5537–5542

Montesano R, Orci L and Vassalli P (1983) In vitro rapid organization of endothelial cells into capillary-like networks is promoted by collagen matrices. *J Cell Biol* **97**: 1648–1652

Pettit GR, Singh SB, Hamel E, Lin CM, Alberts DS and Garcia-Kendall D (1989) Isolation and structure of the strong cell growth and tubulin inhibitor combretastatin A-4. *Experientia* **45**: 209–211

Pettit GR, Temple CJR, Nnarayanan VL, Varma R, Simpson MJ, Boyd MR, Rener GA and Bansal NN (1995) Antineoplastic agent 322. Synthesis of combretastatin A-4 prodrugs. *Anticancer Drug Des* **10**: 299–309

Rewcastle GW, Atwell GJ, Baguley BC, Calveley SB and Denny WA (1989) Potential antitumour agents. 58. Synthesis and structure-activity relationships of substituted xanthenone-4-acetic acids active against the Colon 38 tumour in vivo. *J Med Chem* **32**: 793–799

Rowinsky EK, Cazenave LA and Donehower RC (1990) Taxol: a novel investigational antimicrotubule agent. *J Natl Cancer Inst* **82**: 1247–1259

Schiff PB and Horwitz SB (1980) Taxol stabilizes microtubules in mouse fibroblast cells. *Proc Natl Acad Sci USA* **77**: 1561–1565

Schiff PB, Fant J and Horwitz SB (1979) Promotion of microtubule assembly in vitro by taxol. *Nature* **22**: 665–667

Schor AM, Schor SL and Allen TD (1983) Effects of culture conditions on the proliferation, morphology and migration of bovine aortic endothelial cells. *J Cell Science* **62**: 267–285

Smith KA, Hill SA, Begg AC and Denekamp J (1988). Validation of the fluorescent dye Hoechst 33342 as a vascular space marker in tumours. *Br J Cancer* **57**: 247–253

UKCCCR Guidelines for the Welfare of Animals in Experimental Neoplasia (1998) *Br J Cancer* **77**: 1–10

Vaupel P, Kallinowski F and Okunieff P (1989) Blood flow, oxygen and nutrient supply and metabolic microenvironment of human tumours. A review. *Cancer Res* **49**: 6449–6465

Warren BA (1979) The vascular morphology of tumours. In: *Tumour Blood Circulation: Angiogenesis, Vascular Morphology and Blood Flow of Experimental and Human Tumours,* Peterson H (ed.), pp: 1–47. Franklin, USA

Woods JA, Hadfield JA, Pettit GR, Fox BW and McGown AT (1995) The interaction with tubulin of a series of stilbenes based on combretastatin A-4. *Br J Cancer* **71**: 705–711

*© 1999 Cancer Research Campaign*

## References

[PDF_00711] Baguley B. C., Holdaway K. M., Thomsen L. L., Zhuang L., Zwi L. J. (1991). Inhibition of growth of colon 38 adenocarcinoma by vinblastine and colchicine: evidence for a vascular mechanism.. Eur J Cancer.

[PDF_00714] Bibby M. C., Double J. A., Loadman P. M., Duke C. V. (1989). Reduction of tumor blood flow by flavone acetic acid: a possible component of therapy.. J Natl Cancer Inst.

[PDF_00720] Bibby M. C., Double J. A., Phillips R. M., Quinn P. K. (1991). Flavone acetic acid: is vascular shutdown the crucial mechanism of action.. Int J Radiat Biol.

[PDF_00717] Bibby M. C., Phillips R. M., Double J. A. (1989). Influence of site on the chemosensitivity of transplantable murine colon tumours to flavone acetic acid (LM975, NSC 347512).. Cancer Chemother Pharmacol.

[PDF_00723] Bibby M. C., Phillips R. M., Double J. A., Pratesi G. (1991). Anti-tumour activity of flavone acetic acid (NSC 347512) in mice--influence of immune status.. Br J Cancer.

[PDF_00726] Bibby M. C., Sleigh N. R., Loadman P. M., Double J. A. (1993). Potentiation of EO9 anti-tumour activity by hydralazine.. Eur J Cancer.

[PDF_00730] Borenfreund E., Babich H., Martin-Alguacil N. (1990). Rapid chemosensitivity assay with human normal and tumor cells in vitro.. In Vitro Cell Dev Biol.

[PDF_00733] Bugajski A., Nowogrodzka-Zagórska M., Leńko J., Miodoński A. J. (1989). Angiomorphology of the human renal clear cell carcinoma. A light and scanning electron microscopic study.. Virchows Arch A Pathol Anat Histopathol.

[PDF_00741] Ching L. M., Joseph W. R., Baguley B. C. (1992). Antitumour responses to flavone-8-acetic acid and 5,6-dimethylxanthenone-4-acetic acid in immune deficient mice.. Br J Cancer.

[PDF_00746] Cowen S. E., Bibby M. C., Double J. A. (1995). Characterisation of the vasculature within a murine adenocarcinoma growing in different sites to evaluate the potential of vascular therapies.. Acta Oncol.

[PDF_00749] Dark G. G., Hill S. A., Prise V. E., Tozer G. M., Pettit G. R., Chaplin D. J. (1997). Combretastatin A-4, an agent that displays potent and selective toxicity toward tumor vasculature.. Cancer Res.

[PDF_00755] Double J. A., Ball C. R., Cowen P. N. (1975). Transplantation of adenocarcinomas of the colon in mice.. J Natl Cancer Inst.

[PDF_00752] Double J. A., de Castro L. C. (1978). Chemotherapy of transplantable adenocarcinomas of the colon in mice. II. Development and characterization of an ascitic line.. Cancer Treat Rep.

[PDF_00757] Dvorak H. F., Nagy J. A., Dvorak J. T., Dvorak A. M. (1988). Identification and characterization of the blood vessels of solid tumors that are leaky to circulating macromolecules.. Am J Pathol.

[PDF_00767] Hill S. A., Sampson L. E., Chaplin D. J. (1995). Anti-vascular approaches to solid tumour therapy: evaluation of vinblastine and flavone acetic acid.. Int J Cancer.

[PDF_00770] Jaffe E. A., Nachman R. L., Becker C. G., Minick C. R. (1973). Culture of human endothelial cells derived from umbilical veins. Identification by morphologic and immunologic criteria.. J Clin Invest.

[PDF_00772] Laws A. L., Matthew A. M., Double J. A., Bibby M. C. (1995). Preclinical in vitro and in vivo activity of 5,6-dimethylxanthenone-4-acetic acid.. Br J Cancer.

[PDF_00780] Maciag T., Kadish J., Wilkins L., Stemerman M. B., Weinstein R. (1982). Organizational behavior of human umbilical vein endothelial cells.. J Cell Biol.

[PDF_00783] Mahadevan V., Malik S. T., Meager A., Fiers W., Lewis G. P., Hart I. R. (1990). Role of tumor necrosis factor in flavone acetic acid-induced tumor vasculature shutdown.. Cancer Res.

[PDF_00777] McGown A. T., Fox B. W. (1989). Structural and biochemical comparison of the anti-mitotic agents colchicine, combretastatin A4 and amphethinile.. Anticancer Drug Des.

[PDF_00786] Montesano R., Orci L., Vassalli P. (1983). In vitro rapid organization of endothelial cells into capillary-like networks is promoted by collagen matrices.. J Cell Biol.

[PDF_00789] Pettit G. R., Singh S. B., Hamel E., Lin C. M., Alberts D. S., Garcia-Kendall D. (1989). Isolation and structure of the strong cell growth and tubulin inhibitor combretastatin A-4.. Experientia.

[PDF_00792] Pettit G. R., Temple C., Narayanan V. L., Varma R., Simpson M. J., Boyd M. R., Rener G. A., Bansal N. (1995). Antineoplastic agents 322. synthesis of combretastatin A-4 prodrugs.. Anticancer Drug Des.

[PDF_00795] Rewcastle G. W., Atwell G. J., Baguley B. C., Calveley S. B., Denny W. A. (1989). Potential antitumor agents. 58. Synthesis and structure-activity relationships of substituted xanthenone-4-acetic acids active against the colon 38 tumor in vivo.. J Med Chem.

[PDF_00799] Rowinsky E. K., Cazenave L. A., Donehower R. C. (1990). Taxol: a novel investigational antimicrotubule agent.. J Natl Cancer Inst.

[PDF_00803] Schiff P. B., Fant J., Horwitz S. B. (1979). Promotion of microtubule assembly in vitro by taxol.. Nature.

[PDF_00801] Schiff P. B., Horwitz S. B. (1980). Taxol stabilizes microtubules in mouse fibroblast cells.. Proc Natl Acad Sci U S A.

[PDF_00805] Schor A. M., Schor S. L., Allen T. D. (1983). Effects of culture conditions on the proliferation, morphology and migration of bovine aortic endothelial cells.. J Cell Sci.

[PDF_00808] Smith K. A., Hill S. A., Begg A. C., Denekamp J. (1988). Validation of the fluorescent dye Hoechst 33342 as a vascular space marker in tumours.. Br J Cancer.

[PDF_00813] Vaupel P., Kallinowski F., Okunieff P. (1989). Blood flow, oxygen and nutrient supply, and metabolic microenvironment of human tumors: a review.. Cancer Res.

[PDF_00819] Woods J. A., Hadfield J. A., Pettit G. R., Fox B. W., McGown A. T. (1995). The interaction with tubulin of a series of stilbenes based on combretastatin A-4.. Br J Cancer.

